# Anti-Adipogenic Polyacetylene Glycosides from the Florets of Safflower (*Carthamus tinctorius*)

**DOI:** 10.3390/biomedicines9010091

**Published:** 2021-01-19

**Authors:** Su Cheol Baek, Sang Ah Yi, Bum Soo Lee, Jae Sik Yu, Jin-Chul Kim, Changhyun Pang, Tae Su Jang, Jaecheol Lee, Ki Hyun Kim

**Affiliations:** 1 School of Pharmacy, Sungkyunkwan University, Suwon 16419, Korea; schii513@daum.net (S.C.B.); angelna1023@hanmail.net (S.A.Y.); kosboybs@naver.com (B.S.L.); jsyu@bu.edu (J.S.Y.); 2 Natural Product Informatics Research Center, KIST Gangneung Institute of Natural Products, Gangneung 25451, Korea; jckim@kist.re.kr; 3 School of Chemical Engineering, Sungkyunkwan University, Suwon 16419, Korea; chpang@skku.edu; 4 Department of Medicine, Dankook University, Cheonan, Chungnam 31116, Korea; jangts@dankook.ac.kr

**Keywords:** safflower, *Carthamus tinctorius*, polyacetylene glycosides, 3T3-L1 preadipocytes, AMPK

## Abstract

Safflower (*Carthamus tinctorius*) is an annual herb belonging to the Compositae family; it has a history of use as a food colorant, dye, and medicine in oriental countries. LC-MS-UV-based chemical analysis of extract of the florets of *C. tinctorius* led to the isolation of two new C_10_-polyacetylene glycosides, (8*Z*)-decaene-4,6-diyne-1,10-diol-1-*O*-*β*-d-glucopyranoside (**1**) and (8*S*)-deca-4,6-diyne-1,8-diol-1-*O*-*β*-d-glucopyranoside (**2**), together with five known analogs (**3**–**7**). The structures of the new compounds were determined by using 1D and 2D NMR spectroscopic data and HR-MS data, as well as chemical transformations. Of compounds **1**–**7**, compounds **2**, **3**, and **4** inhibited the adipogenesis of 3T3-L1 preadipocytes, whereas compounds **1** and **6** promoted adipogenesis. Compounds **2**, **3**, and **4** also prevented lipid accumulation through the suppression of the expression of lipogenic genes and the increase of the expression of lipolytic genes. Moreover, compounds **3** and **4** activated AMPK, which is known to facilitate lipid metabolism. Our findings provide a mechanistic rationale for the use of safflower-derived polyacetylene glycosides as potential therapeutic agents against obesity.

## 1. Introduction

Obesity is caused by energy imbalance, which leads to an excessive accumulation of body fat in adipose tissues [1]. The expansion of adipose tissue accompanies the differentiation of preadipocytes residing in adipose tissues to mature adipocytes and the generation and accumulation of lipid droplets in adipocytes [2]. Thus, the identification of compounds preventing adipogenesis and lipogenesis has been considered as an effective strategy for the alleviation of obesity and metabolic diseases. 

*Carthamus tinctorius* L., also known as safflower, is a highly branched and thistle-like annual plant belonging to the Compositae family. *C. tinctorius* has a long history of use as a food colorant, dye, and traditional medicine for the treatment of cardiovascular diseases and gynecological symptoms [3]. The pistil of *C. tinctorius* is well-known as an edible dye agent in various European dishes, including paella, risotto, and pasta [4]. In previous studies, phytochemical investigation of this plant reported the presence of quinochalcones, such as carthamin, safflower yellow, and safflomin, flavonoids, alkaloids, and polyacetylenes [5]. Further pharmacological studies demonstrated that the polyacetylenes from *C. tinctorius* inhibited LPS-induced NO (nitric oxide) release, showing its potential as an agent for treating inflammatory diseases [6]. Recent studies have shown that extracts of safflower inhibited the adipogenesis of 3T3-L1 preadipocytes and alleviated high-fat diet-induced obesity in mice [7,8,9,10]. In addition, treatment with safflower extracts improved metabolic parameters, such as glucose metabolism and lipid profiles, in mice with diet-induced obesity [9,10]. Despite numerous evidences showing the beneficial effects of safflower on metabolism, the exact compounds responsible for the action and the mechanisms underlying their action remain undiscovered. 

**Figure 1:**
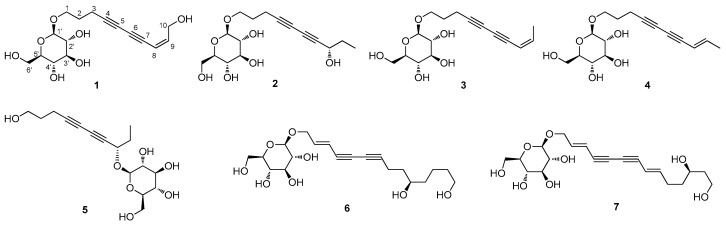
The chemical structures of the isolated compounds **1**–**7**.

As a part of our continued search for natural products with novel structural and/or biological properties [11,12,13,14,15], seven polyacetylene glycosides (**1**–**7**), including two new C_10_-polyacetylene glycosides, (8*Z*)-decaene-4,6-diyne-1,10-diol-1-*O*-*β*-d-glucopyranoside (**1**) and (8*S*)-deca-4,6-diyne-1,8-diol-1-*O*-*β*-d-glucopyranoside (**2**), were isolated from the extract of the florets of *C. tinctorius* by using LC-MS-UV-based chemical analysis. The structures of the new compounds were established by 1D and 2D NMR spectroscopic and high-resolution MS data analysis, and the absolute configurations of the sugar moiety were elucidated by chemical transformations followed by enzymatic hydrolysis. Herein, we have described the isolation and structural characterization of the compounds (**1**–**7**) (Figure 1) and the evaluation of their effects on de novo adipogenesis and lipid metabolism in adipocytes.

## 2. Materials and Methods

### 2.1. General Experimental Procedures

Optical rotations were measured using a Jasco P-2000 polarimeter (Jasco, Easton, MD, USA). IR spectra were recorded using a Bruker IFS-66/S FT-IR spectrometer (Bruker, Karlsruhe, Germany). Ultraviolet (UV) spectra were acquired using an Agilent 8453 UV-visible spectrophotometer (Agilent Technologies, Santa Clara, CA, USA). NMR spectra were recorded using a Bruker AVANCE III HD 850 NMR spectrometer with a 5-mm TCI CryoProbe, operated at 850 MHz (^1^H) and 212.5 MHz (^13^C). The chemical shifts were presented in ppm (*δ*) for ^1^H and ^13^C NMR analyses. Preparative HPLC was performed by using a Waters 1525 Binary HPLC pump with a Waters 996 photodiode array detector (Waters Corporation, Milford, MA, USA) and an Agilent Eclipse C18 column (250 × 21.2 mm, 5 µm; flow rate: 5 mL/min; Agilent Technologies). Semi-preparative HPLC was performed on a Shimadzu Prominence HPLC System with SPD-20A/20AV Series Prominence HPLC UV-Vis detector (Shimadzu, Tokyo, Japan) and a Phenomenex Luna C18 column (250 × 10 mm, 5 µm; flow rate: 2 mL/min; Phenomenex, Torrance, CA, USA). LC-MS analysis was performed using an Agilent 1200 Series HPLC system, equipped with a diode array detector and 6130 Series ESI mass spectrometer, and an analytical Kinetex C18 100 Å column (100 × 2.1 mm, 5 µm; flow rate: 0.3 mL/min; Phenomenex). All HRESIMS data were obtained by using a Waters Xevo G2 quadrupole time-of-flight (QTOF) mass spectrometer and a Synapt G2 HDMS QTOF mass spectrometer (Waters). Silica gel 60 (230–400 mesh; Merck, Darmstadt, Germany) and RP-C_18_ silica gel (Merck, 230–400 mesh) were used for column chromatography. The packing material for molecular sieve column chromatography was Sephadex LH-20 (Pharmacia, Uppsala, Sweden). Thin-layer chromatography was performed by using precoated silica gel F_254_ plates and RP-C_18_ F_254s_ plates (Merck) and spots were detected under UV light or by heating, after spraying with anisaldehyde–sulfuric acid.

### 2.2. Plant Material

The florets of *C. tinctorius* were collected in Pocheon, Gyeonggi-do, Korea, and purchased from Dongyangpharm in September 2018. The plant was identified by one of the authors (K. H. Kim). A voucher specimen (HH-18-12) was deposited in the herbarium of the School of Pharmacy, Sungkyunkwan University, Suwon, Korea. 

### 2.3. Extraction and Isolation

The florets of *C. tinctorius* (1.8 kg) were extracted using 80% aqueous MeOH (each 20 L × 2 days) at room temperature, filtered through Whatman’s filter paper No. 1, and then combined and concentrated under vacuum pressure by using a rotary evaporator, which yielded an MeOH extract (530.0 g). The MeOH extract was suspended in distilled water (700 mL, each) and then solvent-partitioned with hexane, dichloromethane, ethyl acetate, and *n*-butanol, which afforded four fractions: hexane-soluble (37.6 g), CH_2_Cl_2_-soluble (3.3 g), EtOAc-soluble (10.4 g), and BuOH-soluble fractions (57.3 g). After solvent partition, the residue was solvent-partitioned again with acetone, affording the acetone-soluble fraction (68.7 g). All these fractions were initially analyzed by LC-MS, which confirmed the presence of components with a distinctive UV spectrum, which was characteristic of an enediyne chromophore [16,17] in the EtOAc-soluble fraction. To identify the compounds of interest, the EtOAc fraction (10.4 g) was resolved by silica gel column chromatography [300 g; eluted using a CH_2_Cl_2_/MeOH (50:1→1:1, *v*:*v*) gradient solvent system and washed with 90% MeOH] and investigated by monitoring through LC-MS using our in-house UV library. As a result, seven fractions (E1–E7) were obtained from the column chromatography, and fraction E5 (3.2 g) was separated by MPLC, using the Yamazen UNIVERSAL Premium ODS-SM column with MeOH/H_2_O (30–100% MeOH) to yield five subfractions (E51–E55). Subfraction E52 (638.7 mg) was loaded onto a Sephadex LH-20 column by using a solvent system of 100% MeOH to yield three subfractions (E521–E523). Subfraction E521 (267.2 mg) was separated by preparative reversed-phase HPLC using a gradient solvent system of MeOH/H_2_O (50–100% MeOH) to yield five subfractions (E5211–E5215). Subfraction E5212 (87 mg) was subjected to silica gel column chromatography [3.0 g, eluted with CH_2_Cl_2_/MeOH (50:1→1:1) gradient solvent system, and washed with 90% MeOH] to yield five subfractions (E52121–E52125). Subfraction E52125 (20.7 mg) was purified by semi-preparative HPLC (36% MeOH) to furnish compound **5** (*t*_R_ 29.5 min, 3.0 mg). Subfraction E5215 (23.4 mg) was separated by semi-preparative HPLC (17% acetonitrile, MeCN) to yield compound **2** (*t*_R_ 38.0 min, 1.5 mg). Subfraction E522 (68.2 mg) was purified by using semi-preparative HPLC (35% MeOH) to yield compound **1** (*t*_R_ 43.0 min, 1.2 mg). Fraction E6 (1.3 g) was separated by MPLC with a gradient solvent system MeOH/H_2_O (30–100% MeOH) to yield five subfractions (E61–E65). Subfraction E62 (172.7 mg) was subjected to Sephadex LH-20 column using a solvent system of 100% MeOH to yield three subfractions (E621–E623). Subfraction E622 (46.6 mg) was purified by semi-preparative HPLC (37% MeOH) to yield compounds **6** (*t*_R_ 42.5 min, 1.5 mg) and **7** (*t*_R_ 47.5 min, 3.1 mg). Subfraction E64 (299.4 mg) was separated by preparative reversed-phase HPLC with a gradient solvent system of MeOH/H_2_O (70% MeOH) to yield five subfractions (E641–E645). Subfraction E643 (96.2 mg) was purified by semi-preparative HPLC (28% MeCN) to furnish compounds **3** (*t*_R_ 32.5 min, 12.5 mg) and **4** (*t*_R_ 34.0 min, 1.5 mg).

#### 2.3.1. (8*Z*)-Decaene-4,6-diyne-1,10-diol-1-*O*-*β*-d-glucopyranoside (**1**)

Yellowish amorphous gum; ${\left[a\right]}_{D}^{25}$ − 68.0 (c 0.06, MeOH); UV (MeOH) λ_max_ (log ε) 208 (3.41), 215 (3.47), 240 (0.72), 255 (1.46), 268 (1.76), 287 (1.66) nm; IR (KBr) ν_max_ 3396, 2976, 2265, 1655, 1610, 1365, 1116 cm^−1^; ^1^H (850 MHz) and ^13^C (212.5 MHz) NMR data, see Table 1; ESIMS (positive-ion mode) *m*/*z* 349.1 [M + Na]^+^; HRESIMS (positive-ion mode) *m*/*z* 349.1252 [M + Na]^+^ (calculated for C_16_H_22_O_7_Na, 349.1263).

#### 2.3.2. (8*S*)-Deca-4,6-diyne-1,8-diol-1-*O*-*β*-d-glucopyranoside (**2**)

Colorless amorphous gum; ${\left[a\right]}_{D}^{25}$ − 18.5 (c 0.07, MeOH); UV (MeOH) λ_max_ (log ε) 210 (3.03), 222 (3.46), 242 (0.68), 255 (0.98), 270 (1.21), 285 (0.91) nm; IR (KBr) ν_max_ 3374, 2953, 2205, 1650, 1235, 1051 cm^−1^; ^1^H (850 MHz) and ^13^C (212.5 MHz) NMR data, see Table 1; ESIMS (positive-ion mode) *m*/*z* 351.1 [M + Na]^+^; HRESIMS (positive-ion mode) *m*/*z* 329.1598 [M + H]^+^ (calculated for C_16_H_25_O_7_, 329.1600).

### 2.4. Enzymatic Hydrolysis and Absolute Configuration Determination of the Sugar Moiety of ***1*** and ***2***

The absolute configuration of the sugar moiety was determined by using an LC-MS-UV-based method [18]. Compounds **1** and **2** (each 0.5 mg) were hydrolyzed with crude hesperidinase (10 mg, from *Aspergillus niger*; Sigma-Aldrich, Saint Louis, MO, USA) at 37 °C for 72 h, individually, and then, EtOAc was used for the extraction. Each aqueous layer was evaporated by using a vacuum evaporator and dissolved in anhydrous pyridine (0.5 mL) with the addition of L-cysteine methyl ester hydrochloride (1.0 mg). After the reaction mixture was heated at 60 °C for 1 h, *O*-tolylisothiocyanate (50 μL) was added, and the mixture was incubated at 60 °C for 1 h. The reaction product was evaporated by using a vacuum evaporator and dissolved in MeOH. Subsequently, the dissolved reaction product was directly analyzed by LC-MS [MeOH/H_2_O, 1:9→7:3 gradient system (0–30 min), 100% MeOH (31–41 min), 0% MeOH (42–52 min); 0.3 mL/min] using an analytical Kinetex C18 100 Å column (100 mm × 2.1 mm i.d., 5 μm). The sugar moiety from **1** and **2** was identified as d-glucopyranose based on the comparison of the retention time with an authentic sample (*t*_R_: d-glucopyranose 19.3 min).

### 2.5. Cell Culture and Differentiation

3T3-L1 preadipocytes, purchased from the American Type Culture Collection (ATCC^®^ CL-173™, Manassa, VA, USA), were grown in Dulbecco Modified Eagle Medium (DMEM) supplemented with 10% bovine calf serum and 1% penicillin/streptomycin (P/S) [19]. For the adipogenic differentiation, 3T3-L1 cells were incubated in DMEM with 10% fetal bovine serum (FBS), 1% P/S, 0.5 mM 3-isobuyl-1-methylxanthine (IBMX), 1 μM dexamethasone, and 1 μg/mL insulin. Then, the medium was replaced with DMEM containing 10% FBS, 1% P/S, and 1 μg/mL insulin every alternate day until Day 8. To evaluate the effects of compounds **1**–**7** on adipogenesis, we treated 3T3-L1 cells with these compounds throughout the process of adipogenesis.

### 2.6. Oil Red O Staining

To observe the accumulated lipid droplets in adipocytes, Oil Red O staining was performed after the differentiation [20]. After the adipocytes were fixed in 10% formaldehyde for 1 h and washed with 60% isopropanol, mature adipocytes were incubated with the Oil Red O working solution for 1 h. Then, the cells were washed with distilled water twice, and images of the stained lipid droplets were captured with Cytation™ 5. 

### 2.7. Cell Counting

First, 3T3-L1 cells were treated with compounds **1**–**7** at concentrations of 10, 20, and 40 μM for 24 h; then, they were incubated with EDTA for 5 min for detachment. The detached cells were diluted with PBS, and the numbers of cells were counted by using a LUNA-II™ Automated Cell Counter (Logos Biosystems, Annandale, VA, USA).

### 2.8. Western Blotting 

Proteins were extracted with Pro-Prep (Intron Biotechnology, Seoul, Korea) for 20 min on ice and then centrifuged at 13,000 rpm at 4 °C for 20 min. For Western blotting, 15 μg of each protein in the supernatant was separated by SDS-polyacrylamide gel (10%) electrophoresis. The proteins were transferred to polyvinylidene difluoride (PVDF, Millipore, Darmstadt, Germany) membranes using a semi-dry transfer apparatus (Bio-Rad, Hercules, CA, USA). The membranes were incubated with the primary antibodies (dilution 1:2000) overnight at 4 °C, followed by incubation with horseradish peroxidase (HRP)-conjugated secondary antibodies (Abcam) for 1 h at room temperature. HRP signals reacting with chemiluminescence reagents (Abclon) were detected using AGFA X-ray films and quantified using the ImageJ software. Anti-phospho (T172) AMPKα (Abcam, ab2535) and anti-AMPKα (Abcam, ab2532S) antibodies were used for Western blotting.

### 2.9. Reverse Transcription and Quantitative Real-Time PCR (RT-qPCR) 

Total RNA was extracted from adipocytes using Easy-Blue reagent (Intron Biotechnology) in accordance with the manufacturer’s instructions. For reverse transcription (RT), cDNA was synthesized from 1 μg of total RNA using the Maxim RT-PreMix Kit (Intron Biotechnology). For quantitative real-time PCR (qPCR), cDNA from RNA was incubated with KAPA SYBR^®^ FAST qPCR Master Mix (Kapa Biosystems) and primers for each gene. The qPCR reaction was detected using a CFX96 Touch^TM^ real-time PCR detector (Bio-Rad). The relative mRNA expression for each gene was normalized to the expression of *β-actin*. The sequences of qPCR primers used in this study are listed in Table 2.

### 2.10. Statistical Analysis

Statistical significance was analyzed by using a two-tailed Student’s *t*-test with Excel and evaluated by using a *p*-value. The data represent the mean ± SEM for *n* = 3 replicates. * *p* < 0.05, ** *p* < 0.01, and *** *p* < 0.001 vs. the control group.

## 3. Results and Discussion

### 3.1. Isolation of the Compounds

The crude extract of the florets of *C. tinctorius* was solvent-partitioned using water and five organic solvents of increasing polarity (hexane, dichloromethane, ethyl acetate, *n*-butanol, and acetone). The obtained fractions were primarily monitored and analyzed by LC-MS, which allowed us to identify the presence of components with a distinctive UV spectrum in the EtOAc-soluble fraction, which is characteristic of an enediyne chromophore [16,17]. To identify the compounds of interest, the LC-MS-based phytochemical analysis of the EtOAc fraction and preparative and semi-preparative HPLC were performed; the processes resulted in the isolation of two new C_10_-polyacetylene glycosides (**1** and **2**) and five known analogs (**3**–**7**). 

### 3.2. Structural Elucidation of Isolated Compounds

**Figure 2:**
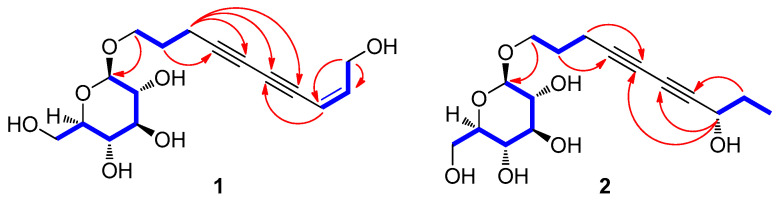
^1^H-^1^H COSY (bold blue lines) and key HMBC (red arrows) correlations of **1** and **2**.

Compound **1** was obtained as yellowish amorphous gum and exhibited UV data characteristic of an enediyne chromophore (Figure S2, Supplementary Materials) [16,17]. The molecular formula was established as C_16_H_22_O_7_ from the molecular ion peak [M + Na]^+^ at *m*/*z* 349.1252 (calculated for C_16_H_22_O_7_Na, 349.1263) in the positive-ion HR-ESIMS (Figure S1) and NMR data (Table 1). The IR spectrum showed absorptions attributable to the hydroxy (3396 cm^−1^) and acetylenic (2265 cm^−1^) groups. The ^1^H (Figure S3) and ^13^C NMR spectra of **1** (Table 1), combined with heteronuclear single quantum coherence (HSQC) and heteronuclear multiple bond correlation (HMBC) data, indicated the presence of a *cis*-configured Δ^8,9^ double bond with signals at *δ*_H_ 5.61 (1H, d, *J* = 11.0 Hz)/*δ*_C_ 109.3 and 6.20 (1H, dt, *J* = 11.0, 6.5 Hz)/*δ*_C_ 146.6, four acetylenic carbons at *δ*_C_ 65.5, 71.4, 80.3, and 85.9, and two oxygenated methylene signals at *δ*_H_ 3.98 (1H, dt, *J* = 10.0, 6.0 Hz) and 3.67 (1H, dt, *J* = 10.0, 6.0 Hz)/*δ*_C_ 68.9 and 4.32 (2H, d, *J* = 6.5 Hz)/*δ*_C_ 60.8, as well as a *β*-d-glucopyranosyl moiety at *δ*_H_ 4.28 (1H, d, *J* = 8.0 Hz)/*δ*_C_ 104.2 and *δ*_C_ 62.5, 71.3, 74.9, 77.7, and 77.8. Detailed NMR analysis and 2D NMR experiments (Figures S4–S6) revealed the strong similarity of **1** with bidenoside C [21], which was isolated as compound **3** in this study. The noticeable difference between the two compounds was that the NMR chemical shifts of C-10 in **1** showed a downfield shift owing to hydroxylation, which was confirmed based on the HMBC correlations between H-10 and C-8/C-9 (Figure 2). The gross structure of **1** was further established by the analysis of cross-peaks in the ^1^H-^1^H COSY (Figure S4) and HMBC (Figure S6) spectra (Figure 2). The absolute configuration of the sugar unit was determined by using an LC-MS-UV-based method [18], and enzymatic hydrolysis of **1** with hesperidinase resulted in the production of a glucopyranose. The *β*-d-glucopyranose was determined by comparing the retention time of its thiocarbamoyl-thiazolidine derivative with that of the standard sample of d-glucopyranose by LC-MS analysis and the coupling constant (*J* = 8.0 Hz) of the anomeric proton signal. Accordingly, the structure of **1** was characterized as (8*Z*)-decaene-4,6-diyne-1,10-diol-1-*O*-*β*-d-glucopyranoside.

**Figure 3:**
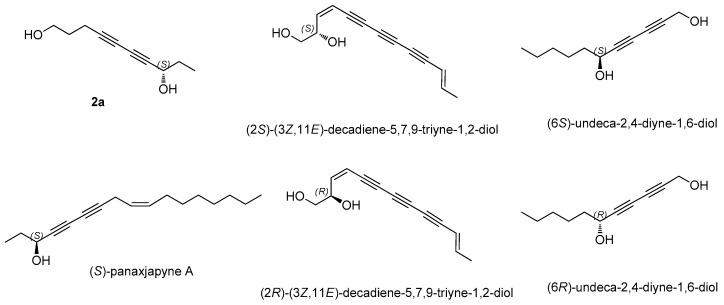
Structures of compound **2a** and the related analogs.

Compound **2** was isolated as a colorless amorphous gum, and its IR spectrum showed absorption bands for hydroxy (3374 cm^−1^) and acetylenic (2205 cm^−1^) groups. Its molecular formula, C_16_H_14_O_7_, was established on the basis of the positive-ion HRESIMS peak at *m*/*z* 329.1598 [M + H]^+^ (calculated for C_16_H_25_O_7_, 329.1600, Figure S7) and NMR data (Table 1). The UV spectrum (Figure S8) of **2** was typical for an enediyne chromophore [16,17]. The ^1^H (Figure S9) and ^13^C NMR spectra of **2** (Table 1) showed a close resemblance to those of 8*S*-deca-4,6-diyne-1,8-diol-8-*O*-*β*-d-glucopyranoside [22], which was isolated as compound **5** in this study. The only slight difference in their chemical shifts was at the level of the glycosylated carbon group at C-1 and C-8, which was observed in compound **2** at *δ*_C_ 69.1 (C-1, Δ*δ*_C_ +7.8 ppm in CD_3_OD) and *δ*_C_ 64.0 (C-8, Δ*δ*_C_ −5.6 ppm in CD_3_OD) [22]. This set of data indicated that compound **2** had a glucose moiety at C-1, the position of which was confirmed by the HMBC correlation between H-1′ and C-1 (Figure 2). The complete structure of **2** was determined further by the 2D NMR analysis [^1^H-^1^H COSY (Figure S10), HSQC (Figure S11), and HMBC (Figure S12)] (Figure 2). The assignment of *β*-d-glucopyranose was also achieved by the LC-MS-UV-based method [18], which was followed by the enzymatic hydrolysis of **2** with hesperidinase. The absolute configuration of C-8 was established by the value of specific rotation of **2a** (aglycone of **2**, 4,6-decadiyne-1,8-diol) derived from the enzymatic hydrolysis of **2**. The specific rotation value of **2a** [${\left[a\right]}_{D}^{25}$ + 12.5 (c 0.01, MeOH)] was comparable with those of the related analogs, (*S*)-panaxjapyne A [${\left[a\right]}_{D}^{28}$ + 11.0 (c 0.1, MeOH)] [23], (2*S*)-(3*Z*,11*E*)-decadiene-5,7,9-triyne-1,2-diol [${\left[a\right]}_{D}^{25}$ + 15.8 (c 0.1, MeOH)] [24], (6*S*)-undeca-2,4-diyne-1,6-diol [${\left[a\right]}_{D}^{25}$ + 10.74 (c 0.37, CH_2_Cl_2_)] [25], (6*R*)-undeca-2,4-diyne-1,6-diol [${\left[a\right]}_{D}^{25}$ − 10.16 (c 0.13, CH_2_Cl_2_)] [25], and (2*R*)-(3*E*,11*Z*)-decadiene-5,7,9-triyne-1,2-diol [${\left[a\right]}_{D}^{25}$ − 16.5 (c 0.1, MeOH)] [24] (Figure 3), which led to the assignment of the absolute configuration of C-8 as *S*. Consequently, the structure of **2** was designated as (8*S*)-deca-4,6-diyne-1,8-diol-1-*O*-*β*-d-glucopyranoside. 

The five known compounds were identified as bidenoside C (**3**) [21], (8*E*)-decaene-4,6-diyne-1-ol-1-*O*-*β*-d-glucopyranoside (**4**) [26], 8*S*-deca-4,6-diyne-1,8-diol-8-*O*-*β*-d-glucopyranoside (**5**) [22], (2*E*)-tetradecaene-4,6-diyne-1,10,14-triol-1-*O*-*β*-d-glucopyranoside (**6**) [6], and (2*E*,8*E*)-tetradecadiene-4,6-diyne-1,12,14-triol-1-*O*-*β*-d-glucopyranoside (**7**) [6] through the comparison of their respective NMR data with previously reported data, in addition to LC-MS analysis.

### 3.3. Evaluation of Biological Activity of the Isolated Compounds

**Figure 4:**
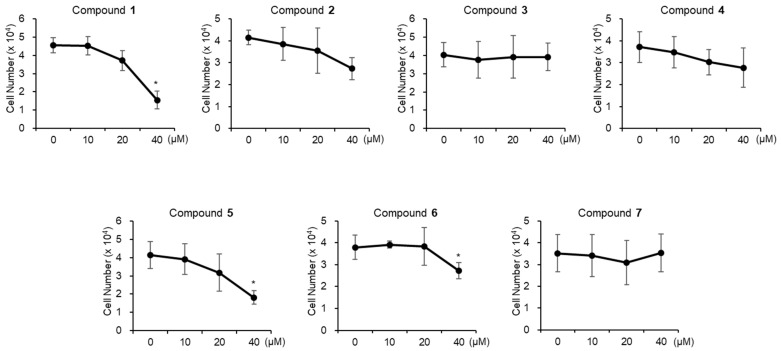
Cytotoxicity of compounds **1**–**7**. Viability of 3T3-L1 cells treated with compounds **1**–**7** (10, 20, and 40 μM) for 24 h was determined by counting the cell number. The data are presented as the mean ± SD for *n* = 3 replicates. * *p* < 0.05.

**Figure 5:**
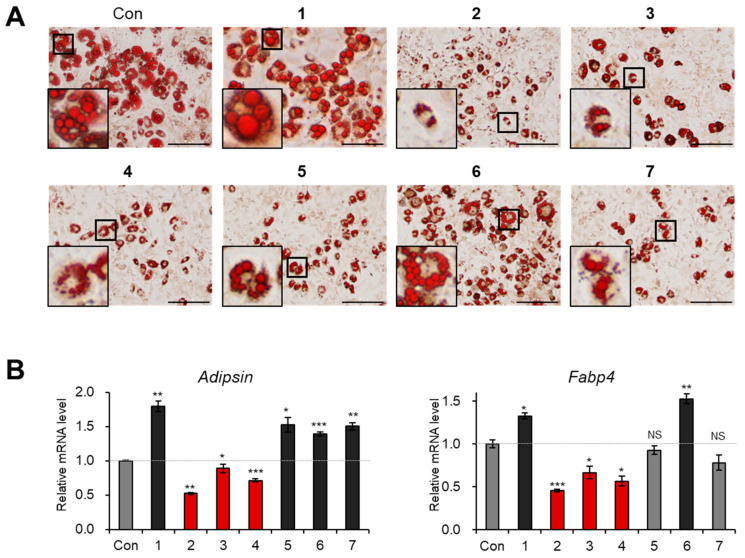
The inhibitory effects of compounds **1**–**7** on adipogenesis. (**A**) The Oil Red O staining of 3T3-L1 adipocytes incubated with compounds **1**–**7** (20 μM) during adipogenesis. Scale bar: 200 μm. 3× magnified images are indicated as black box. (**B**) The relative mRNA expression of *Adipsin* and *Fabp4* in 3T3-L1 adipocytes incubated with compounds **1**–**7** (20 μM) during adipogenesis. The data are presented as the mean ± SEM for *n* = 3 replicates. * *p* < 0.05, ** *p* < 0.01, *** *p* < 0.001 and NS: not significant.

Prior to the assessment of the anti-adipogenic properties of the isolated compounds, we first evaluated the cytotoxic effects of compounds **1**–**7** in 3T3-L1 preadipocytes. No compounds exhibited cytotoxicity at concentrations of 10 and 20 μM, but compounds **1**, **5**, and **6** decreased cell viability at a concentration of 40 μM (Figure 4). Hence, we treated 3T3-L1 cells with compounds **1**–**7** at a concentration of 20 μM during the entire process of adipogenesis for the evaluation of the anti-adipogenic activities of these compounds. Oil red O staining data showed that compounds **2**–**4** prevented the de novo generation of adipocytes and lipid accumulation within adipocytes (Figure 5A). The transcription levels of mature adipocyte marker genes (*Adipsin* and *Fabp4*) were significantly reduced by treatment with compounds **2**–**4** (Figure 5B). These data indicated that the new C_10_-polyacetylene glycoside (**2**) and two known analogs (**3** and **4**) inhibited the adipogenesis of 3T3-L1 preadipocytes.

**Figure 6:**
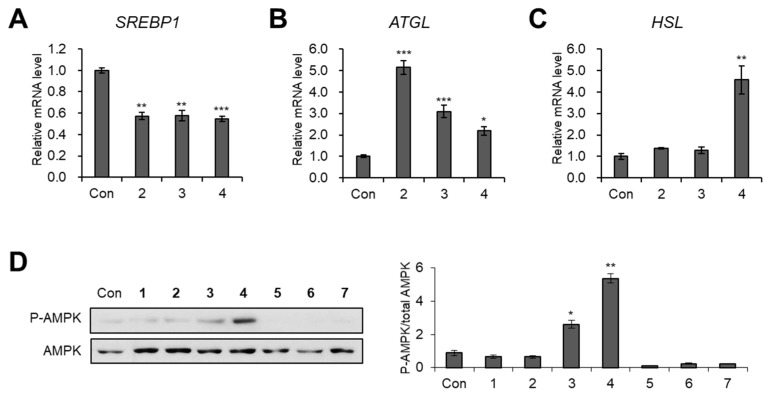
The inhibitory effects of compounds **1**–**7** on lipid metabolism. (**A**–**C**) The relative mRNA expression of *SREBP1* (**A**), *ATGL* (**B**), and *HSL* (**C**) in 3T3-L1 adipocytes incubated with compounds **1**–**7** (20 μM) during adipogenesis. (**D**) Immunoblotting analysis of 3T3-L1 cells incubated with compounds **1**–**7** (20 μM) for 24 h. The data are presented as the mean ± SEM for *n* = 3 replicates. * *p* < 0.05, ** *p* < 0.01, and *** *p* < 0.001.

As the failure of lipid fusion was observed in the groups treated with compounds **2**–**4** (Figure 5A), we then investigated whether compounds **2**–**4** affected lipid metabolism. Upon exposure to compounds **2**–**4** during adipogenesis, the expression of the lipogenic gene *SREBP1* was suppressed (Figure 6A), whereas the mRNA expression of the lipolytic gene *ATGL* was increased (Figure 6B). In the case of compound **4**, the expression of another lipolytic gene, *HSL*, was also markedly enhanced (Figure 6C). These results suggested that compounds **2**–**4** can enhance lipid metabolism through the inhibition of lipogenesis and the facilitation of lipolysis.

AMP-activated protein kinase (AMPK) is known to be a key controller of energy metabolism through the inhibition of adipogenesis and the stimulation of lipid metabolism upon its activation [27]. It has been reported that treatment of brain cells with safflower yellow B derived from *C. tinctorius* induced the phosphorylation of AMPK [28]. Thus, we assessed the effects of compounds **1**–**7** on AMPK activation by detecting the level of phosphorylated AMPK. Western blotting data showed that compounds **3** and **4** significantly increased the phosphorylation of AMPK compared with the total amounts of AMPK (Figure 6D).

## 4. Conclusions

**Figure 7:**
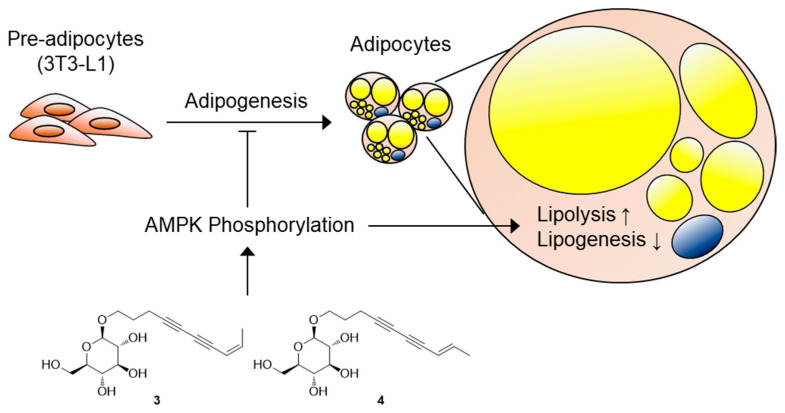
Molecular model explaining the mechanism of action of compounds **3** and **4**. Compounds **3** and **4** inhibited the adipogenesis of 3T3-L1 preadipocytes, induced AMP-activated protein kinase (AMPK) phosphorylation, and stimulated lipid metabolism through the enhancement of lipolysis and the suppression of lipogenesis.

In this study, we identified two novel C_10_-polyacetylene glycosides (**1** and **2**) and five known compounds (**3**–**7**) from the MeOH extract of the florets of *C. tinctorius*. The effects of the seven identified compounds on adipogenesis were evaluated; compounds **2**–**4** efficiently inhibited adipocyte differentiation from 3T3-L1 preadipocytes, reducing the mRNA expression levels of *Adipsin* and *Fabp4*. Furthermore, compounds **2**–**4** promoted the expression of lipolytic genes while downregulating the expression of lipogenic genes. Compounds **3** and **4** induced AMPK phosphorylation, which is known to improve energy metabolism (Figure 7); in contrast, compound **2** appeared to regulate lipid metabolism through other pathways not involved in AMPK signaling. Our findings provide experimental evidence to support the metabolic role of safflower-derived polyacetylene glycosides in the prevention of excessive lipid accumulation in obesity.

## Figures and Tables

**Table 1:** ^1^H and ^13^C NMR data for compounds **1** and **2** in CD_3_OD. ^a^

Position	1		2	
	*δ*_C_, Type	*δ*_H_ (*J* in Hz)	*δ*_C_, Type	*δ*_H_ (*J* in Hz)
1	68.9, CH_2_	3.98, dt (10.0, 6.0) 3.67 dt (10.0, 6.0)	69.1, CH_2_	3.95, dt (10.0, 6.0) 3.64 dt (10.0, 6.0)
2	29.5, CH_2_	1.87, m	29.6, CH_2_	1.83, m
3	16.5, CH_2_	2.52, t (7.0)	16.3, CH_2_	2.44, t (7.0)
4	85.9, C		81.4, C	
5	65.5, C		65.6, C	
6	80.3, C		70.1, C	
7	71.4, C		78.0, C	
8	109.3, CH	5.61, d (11.0)	64.0, CH	4.27, d (6.5)
9	146.6, CH	6.20, dt (11.0, 6.5)	31.7, CH_2_	1.67, m
10	60.8, CH_2_	4.32, d (6.5)	9.6, CH_3_	0.98, t (7.5)
1′	104.2, CH	4.28, d (8.0)	104.5, CH	4.25, d (8.0)
2′	74.9, CH	3.19, dd (9.0, 8.0)	74.9, CH	3.16, dd (9.0, 8.0)
3′	77.7, CH	3.28, m	77.7, CH	3.28, m
4′	71.3, CH	3.29, m	71.3, CH	3.29, m
5′	77.8, CH	3.36, m	77.8, CH	3.35, m
6′	62.5, CH_2_	3.89, dd (12.0, 2.0) 3.69, dd (12.0, 5.5)	62.6, CH_2_	3.86, dd (12.0, 2.0) 3.67, dd (12.0, 5.5)

^a^ Signal multiplicity and coupling constants (Hz) are in parentheses; the measurements are based on HSQC, HMBC, and ^1^H-^1^H COSY experiments.

**Table 2:** Sequences of primers used for RT-qPCR.

Gene	Forward	Reverse
*β-Actin*	5′-ACGGCCAGGTCATCACTATTG-3’	5′-TGGATGCCACAGGATTCCA-3′
*Adipsin*	5′-CATGCTCGGCCCTACATG-3’	5′-CACAGAGTCGTCATCCGTCAC-3′
*Fabp4*	5′-AAGGTGAAGAGCATCATAACCCT-3’	5′-TCACGCCTTTCATAACACATTCC-3′
*SREBP1*	5′-AACGTCACTTCCAGCTAGAC-3’	5′-CCACTAAGGTGCCTACAGAGC-3′
*ATGL*	5′-TTCACCATCCGCTTGTTGGAG-3’	5′-AGATGGTCACCCAATTTCCTC-3′
*HSL*	5′-CACAAAGGCTGCTTCTACGG-3’	5′-GGAGAGAGTCTGCAGGAACG-3′

## Data Availability

Not applicable.

## Supplementary Materials

The following are available online at https://www.mdpi.com/2227-9059/9/1/91/s1.
