# Supplementary material for: Anti-Adipogenic Polyacetylene Glycosides from the Florets of Safflower (Carthamus tinctorius)
Source: Biomedicines. 2021 Jan 19;9(1):91. doi: 10.3390/biomedicines9010091 (PMC7833391; doi:10.3390/biomedicines9010091)

Supplementary data

Anti-adipogenic Polyacetylene Glycosides from the Florets of Safflower (*Carthamus tinctorius*)

Su Cheol Baek [1,a], Sang Ah Yi [1,a], Bum Soo Lee [1], Jae Sik Yu [1], Jin-Chul Kim [2], Changhyun Pang [3], Tae Su Jang [4], Jaecheol Lee [1,*], and Ki Hyun Kim [1,*]

[1]School of Pharmacy, Sungkyunkwan University, Suwon 16419, Republic of Korea; schii513@daum.net (S.C.B); angelna1023@hanmail.net (S.A.Y.); kosboybs@naver.com (B.S.L.); jsyu@bu.edu (J.S.Y.)

[2]KIST Gangneung Institute of Natural Products, Natural Product Informatics Research Center, Gangneung, 25451, Republic of Korea; jckim@kist.re.kr (J.C.K.)

[3]School of Chemical Engineering, Sungkyunkwan University, Suwon 16419, Republic of Korea; chpang@skku.edu (C.P.)

[4]Department of Medicine, Dankook University, Cheonan, Chungnam 31116, Republic of Korea; jangts@dankook.ac.kr (T.S.J.)

[*]Correspondence: Ki Hyun Kim: (Tel: +82-31-290-7700; Fax: +82-31-290-7730; E-mail: khkim83@skku.edu)

Jaecheol Lee: (Tel: +82-31-290-7726; E-mail: jaecheol@skku.edu)

Supporting Information Contents:

Figure S1. The HRESIMS data of **1** ……..……………………..…………………………………………………………………………S3
Figure S2. The UV spectrum of **1**………………..…………………………………………………………...……………………………………S4
Figure S3. The ^{1}H NMR spectrum of **1** (CD_3OD, 850 MHz)……………………...…………………………………………………………S5
Figure S4. The ^{1}H-^{1}H COSY spectrum of **1**…………………………………….………………………………………………………S6
Figure S5. The HSQC spectrum of **1**………S7
Figure S6. The HMBC spectrum of **1**…………………...……………………………………………..……………………………………S8
Figure S7. The HRESIMS data of **2** ……..……………………..…………………………………………………………………………S9
Figure S8. The UV spectrum of **2**………………..…………………………………………………………...…………………………………S10
Figure S9. The ^{1}H NMR spectrum of **2** (CD_3OD, 850 MHz)……………………...……………………………………………………S11
Figure S10. The ^{1}H-^{1}H COSY spectrum of **2**…………………………………….…………………………………………………S12
Figure S11. The HSQC spectrum of **2**……S13
Figure S12. The HMBC spectrum of **2**…………………...……………………………………………..…………………………………S14

Figure S1. The HRESIMS data of **1**

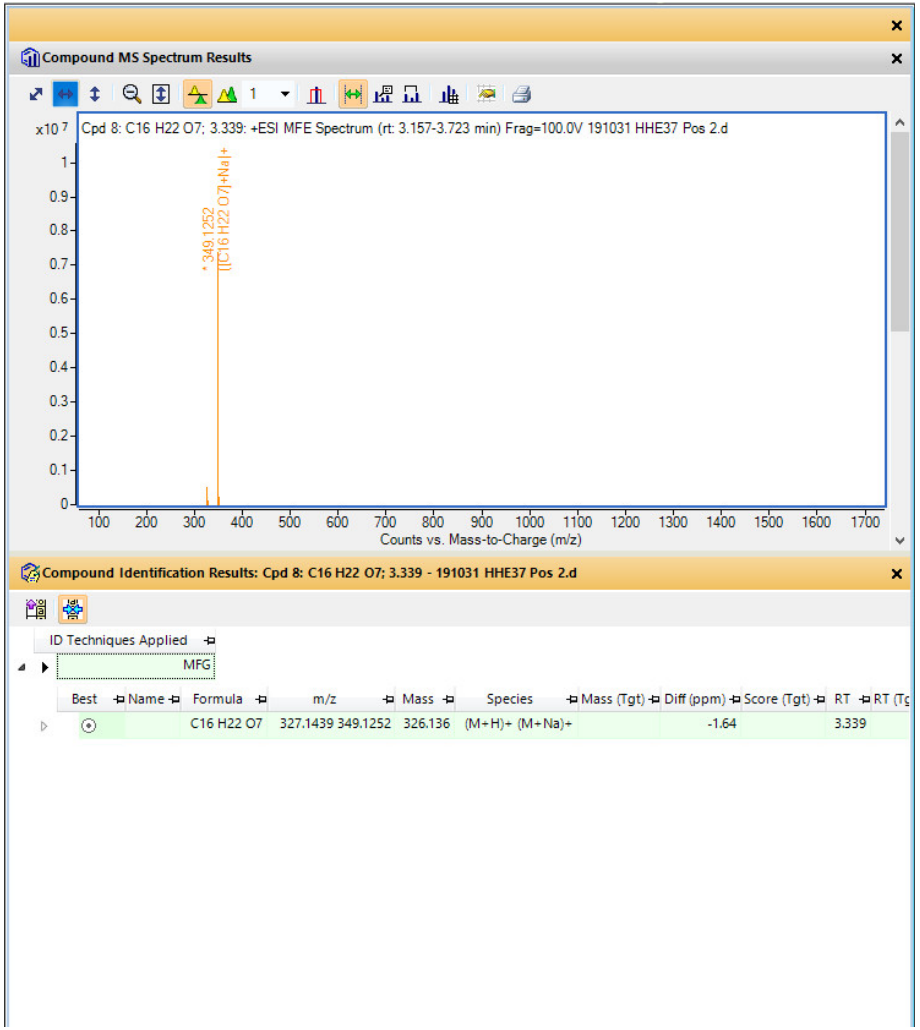

Figure S2. The UV spectrum of **1**

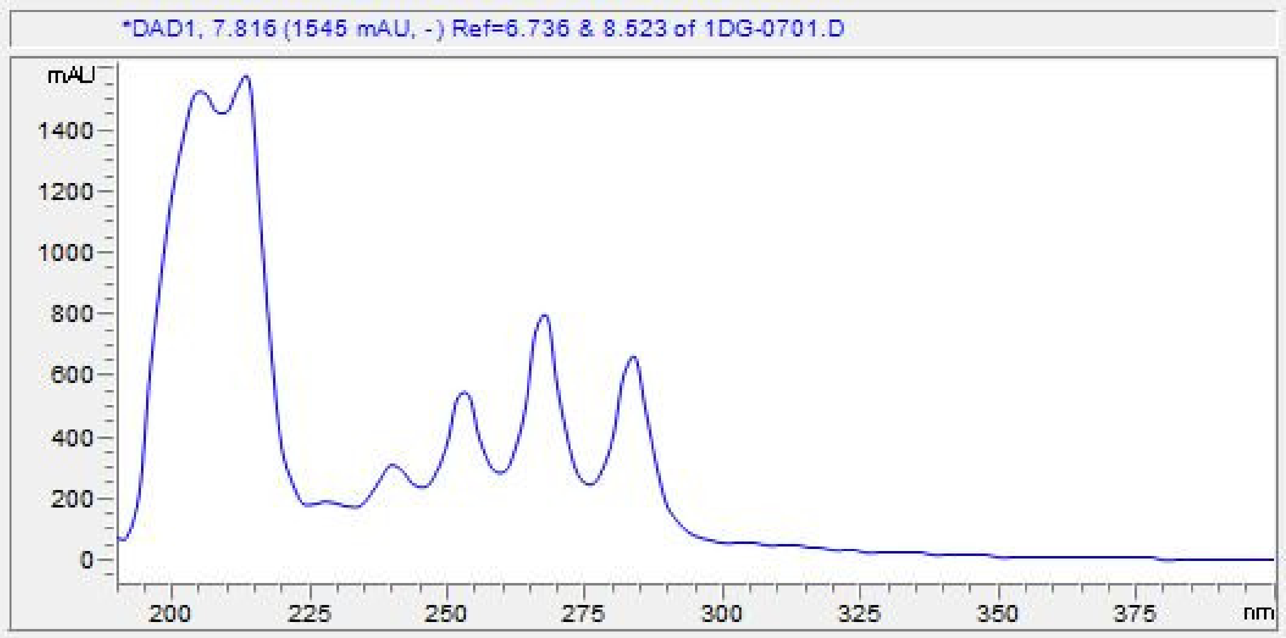

Figure S3. The ^{1}H NMR spectrum of **1** (CD_3OD, 850 MHz)

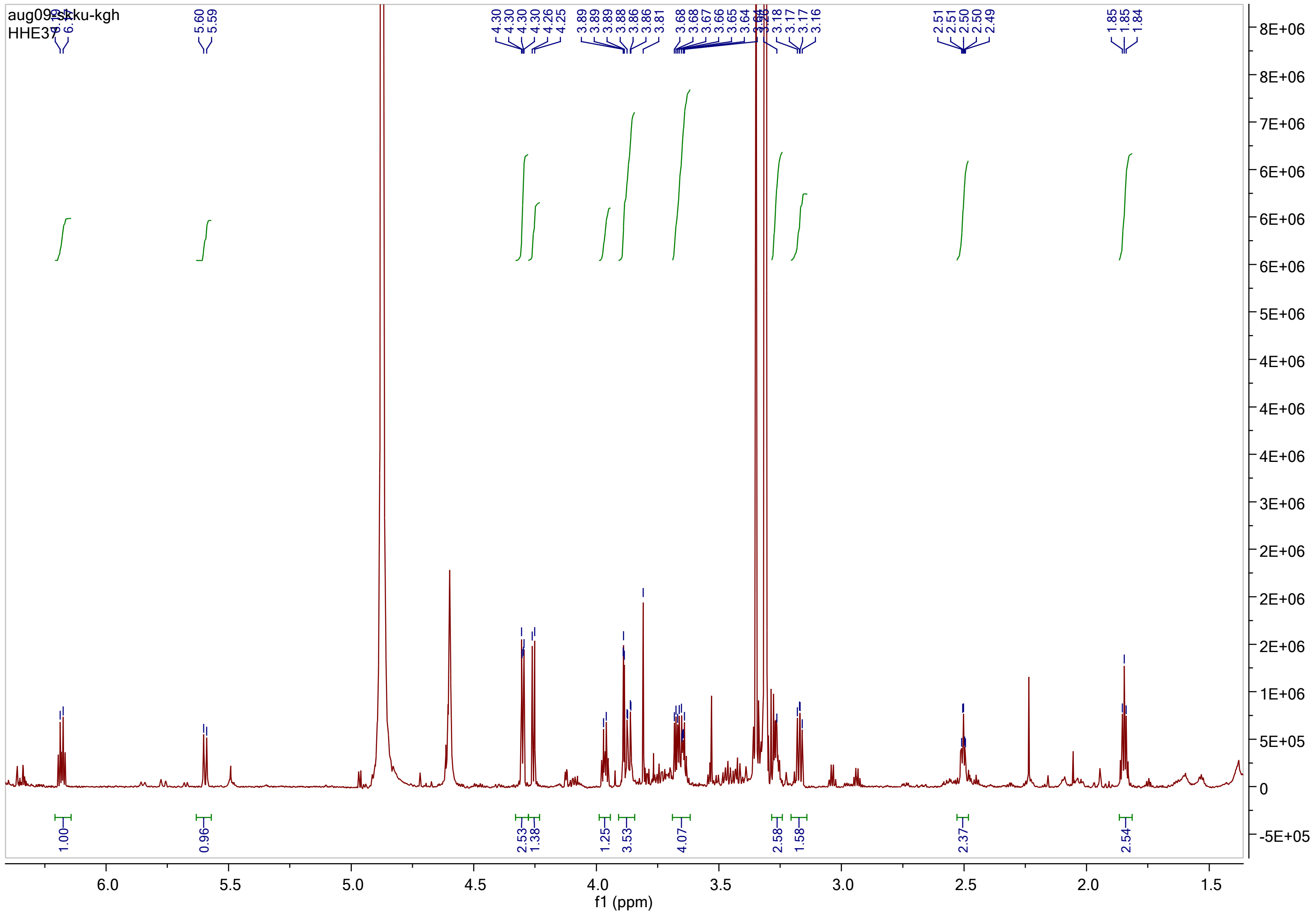

Figure S4. The ^{1}H-^{1}H COSY spectrum of **1**

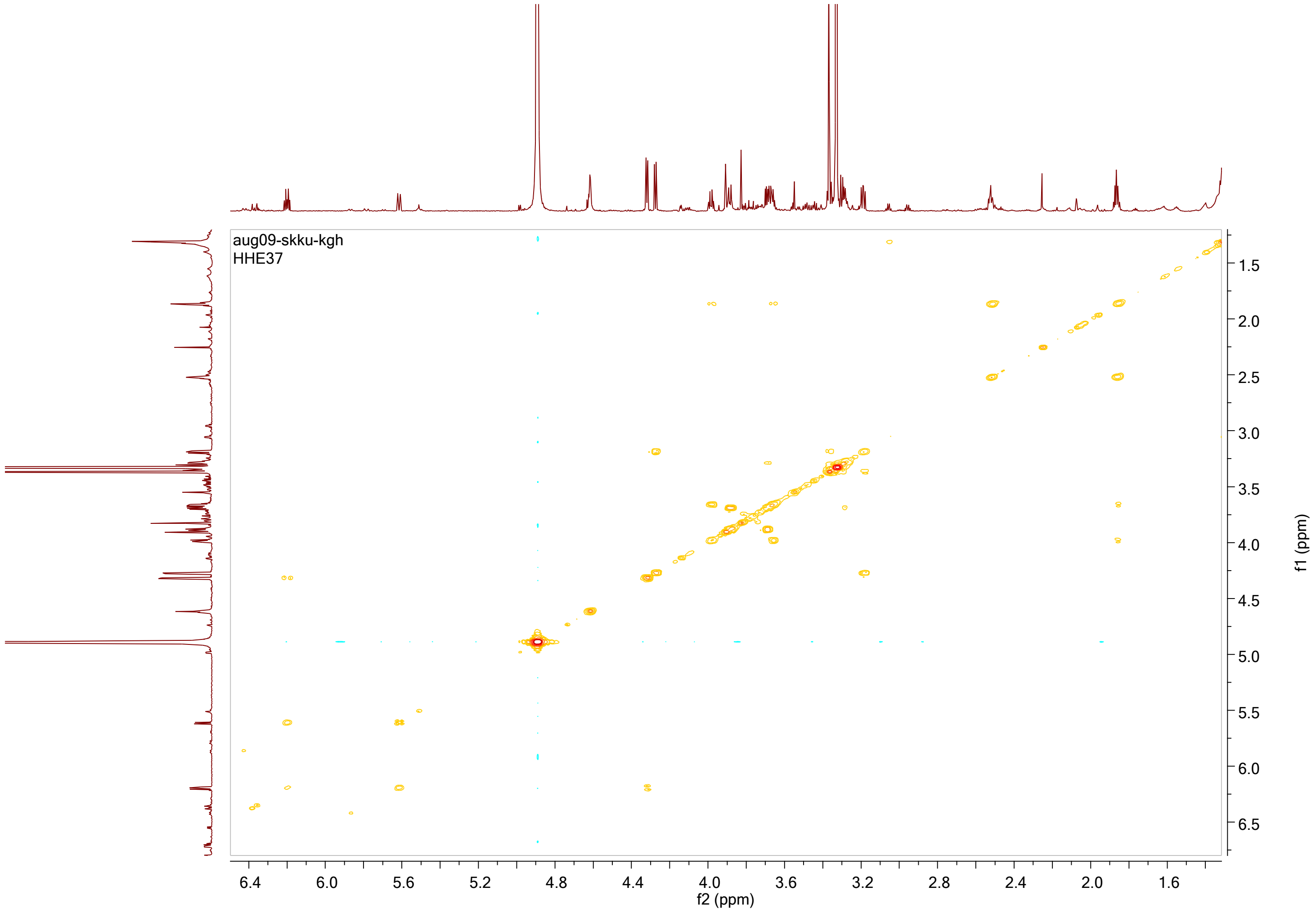

Figure S5. The HSQC spectrum of **1**

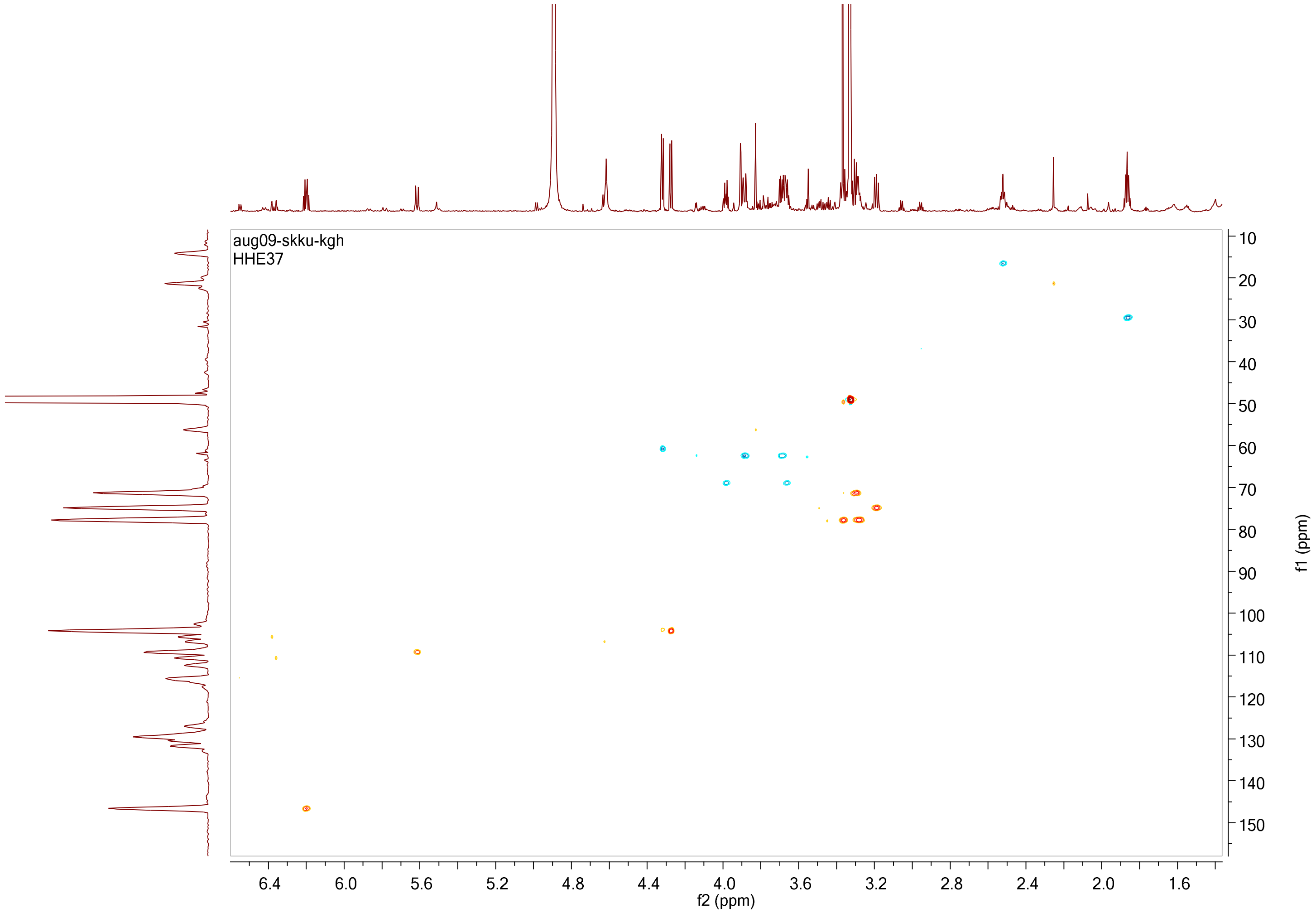

Figure S6. The HMBC spectrum of **1**

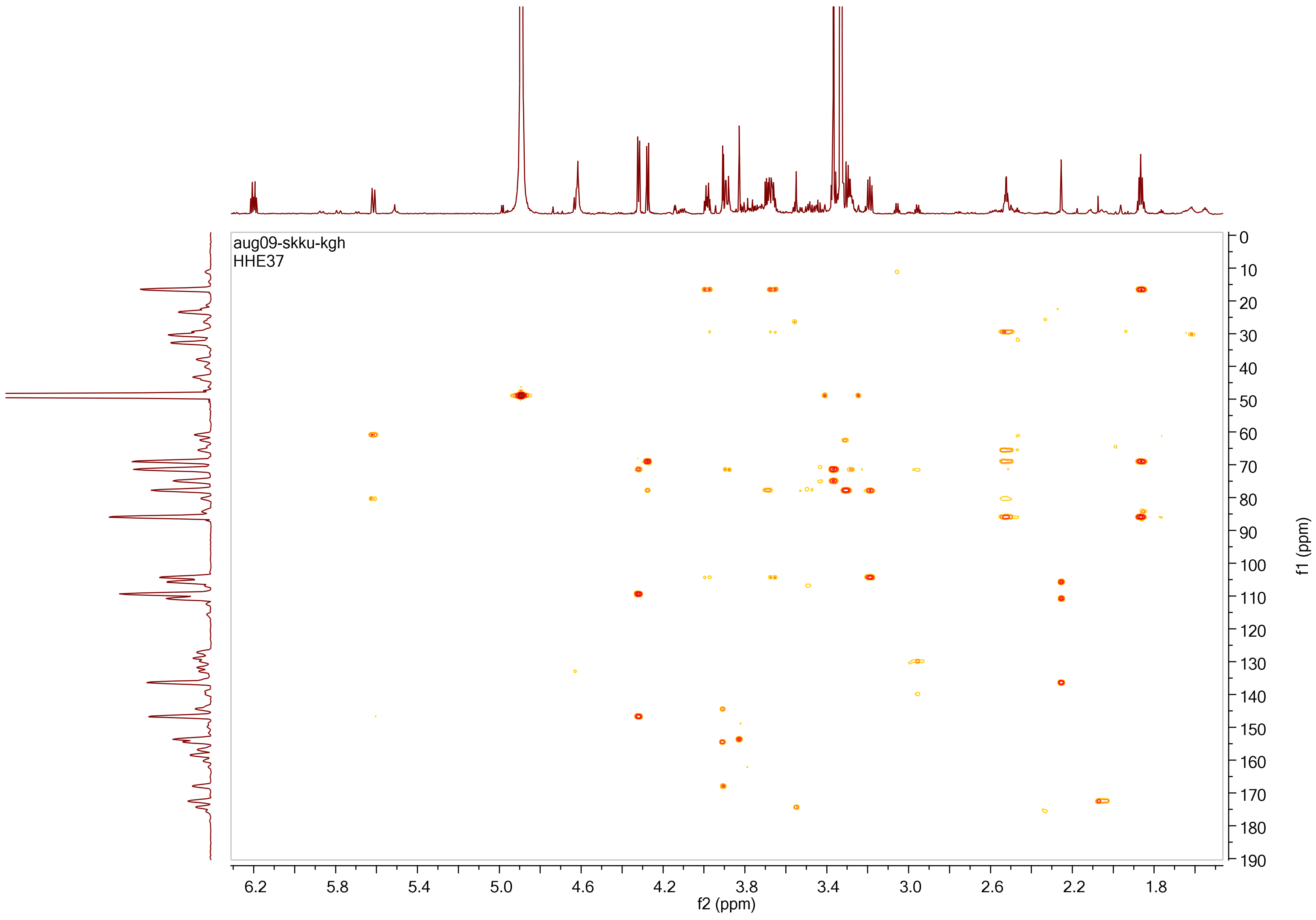

Figure S7. The HRESIMS data of **2**

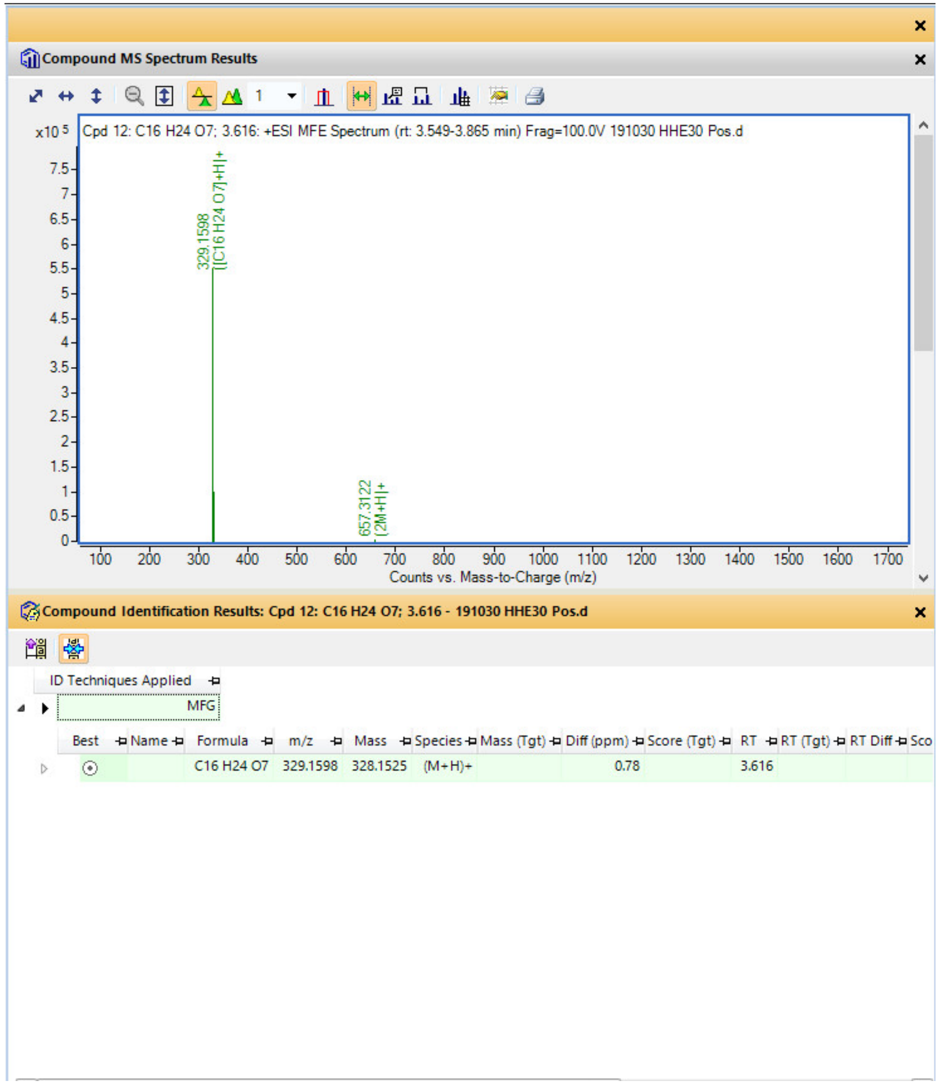

Figure S8. The UV spectrum of **2**

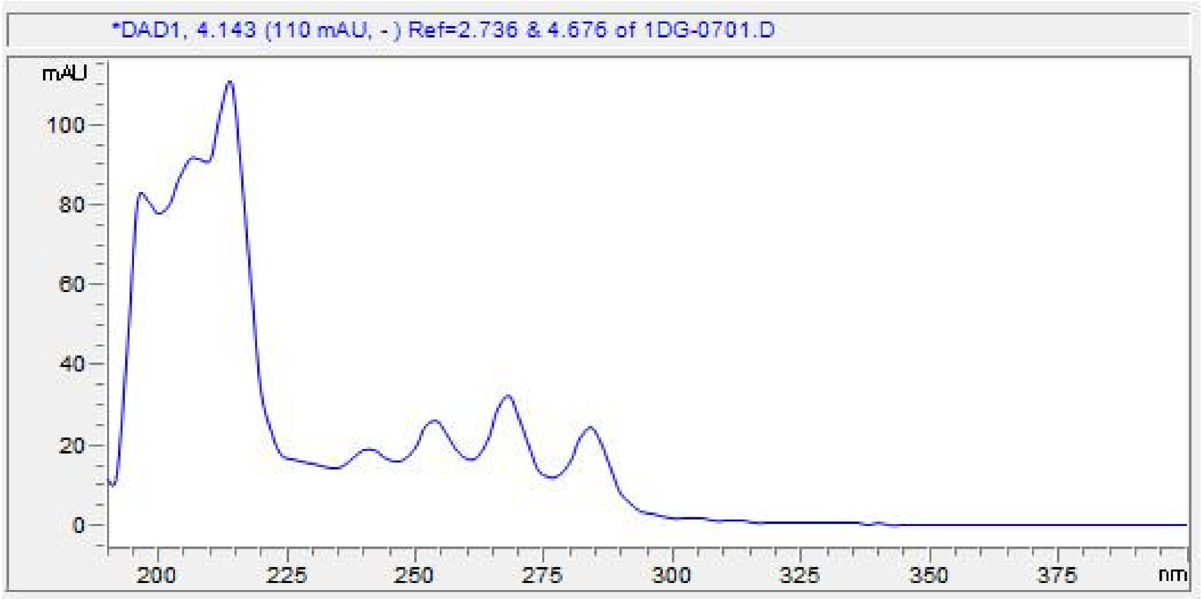

Figure S9. The ^{1}H NMR spectrum of **2** (CD_3OD, 850 MHz)

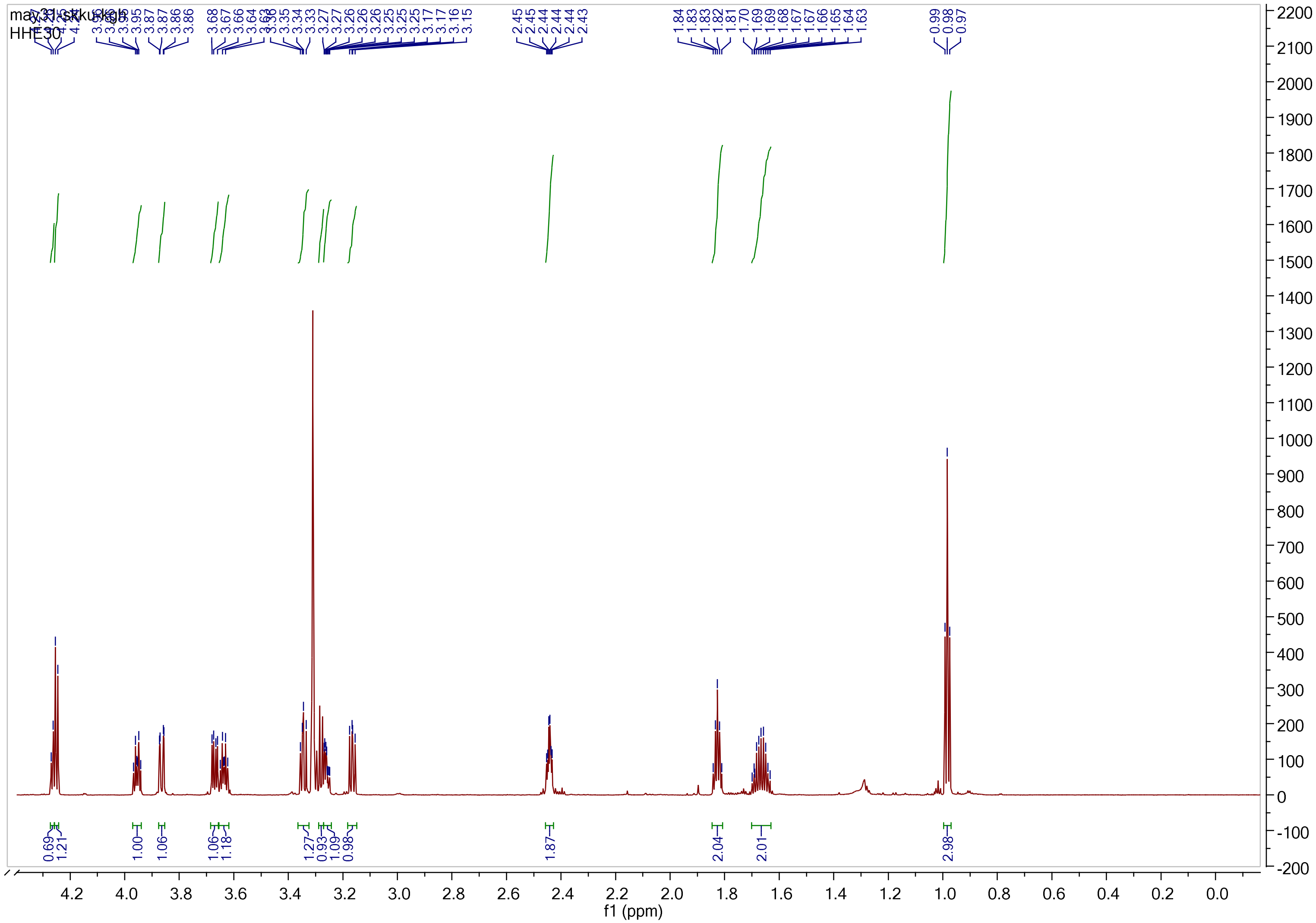

Figure S10. The ^{1}H-^{1}H COSY spectrum of **2**

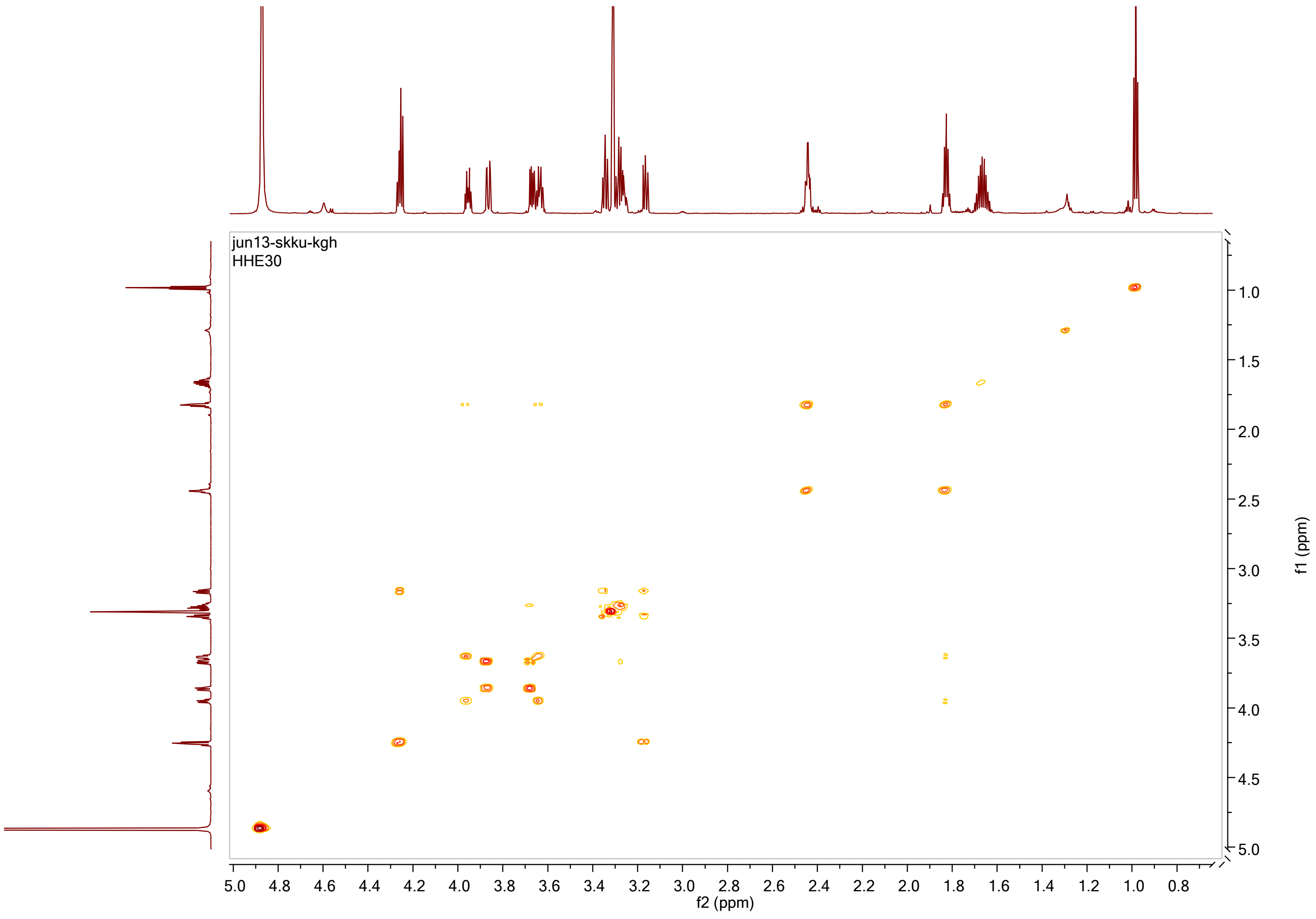

Figure S11. The HSQC spectrum of **2**

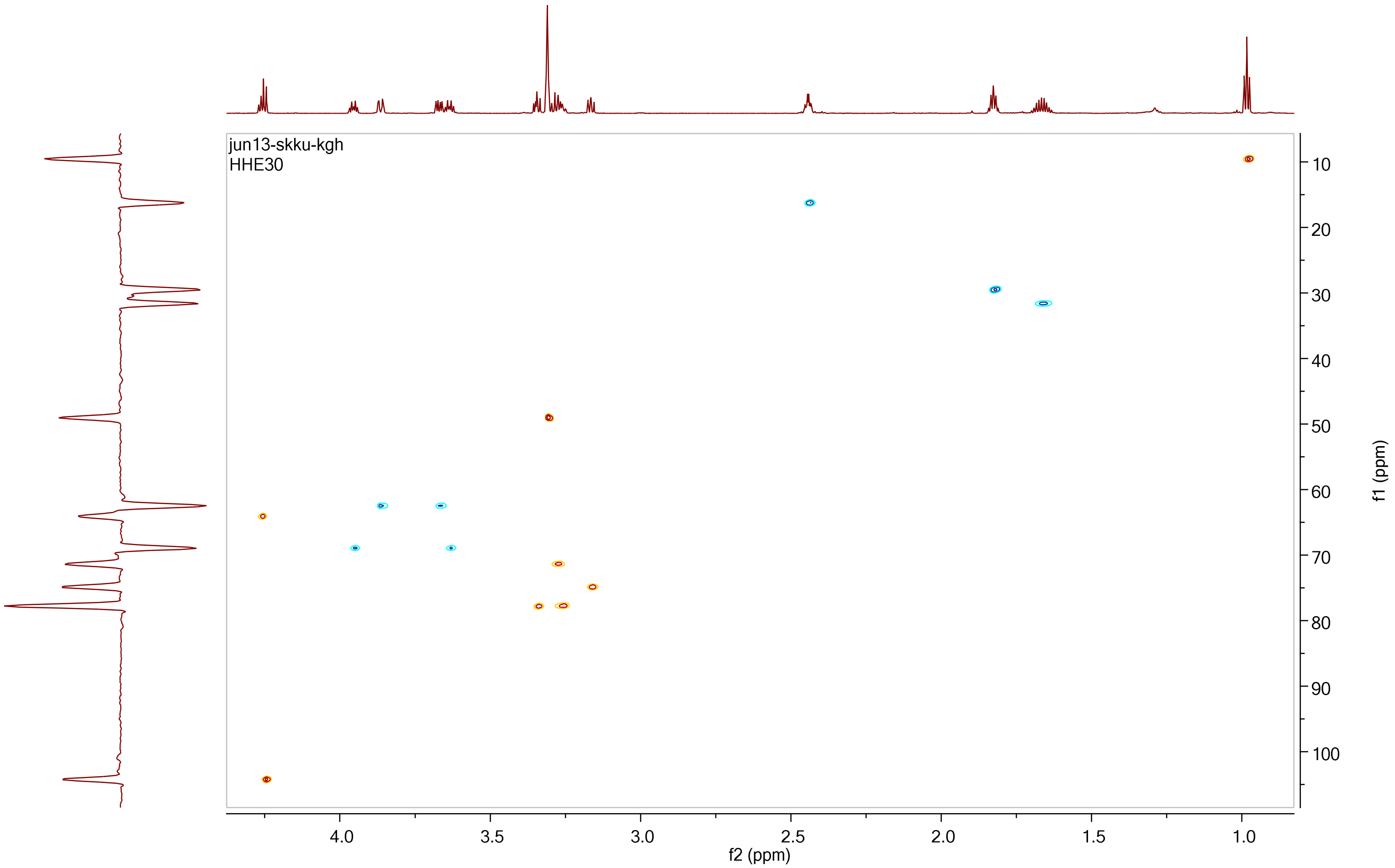

Figure S12. The HMBC spectrum of **2**

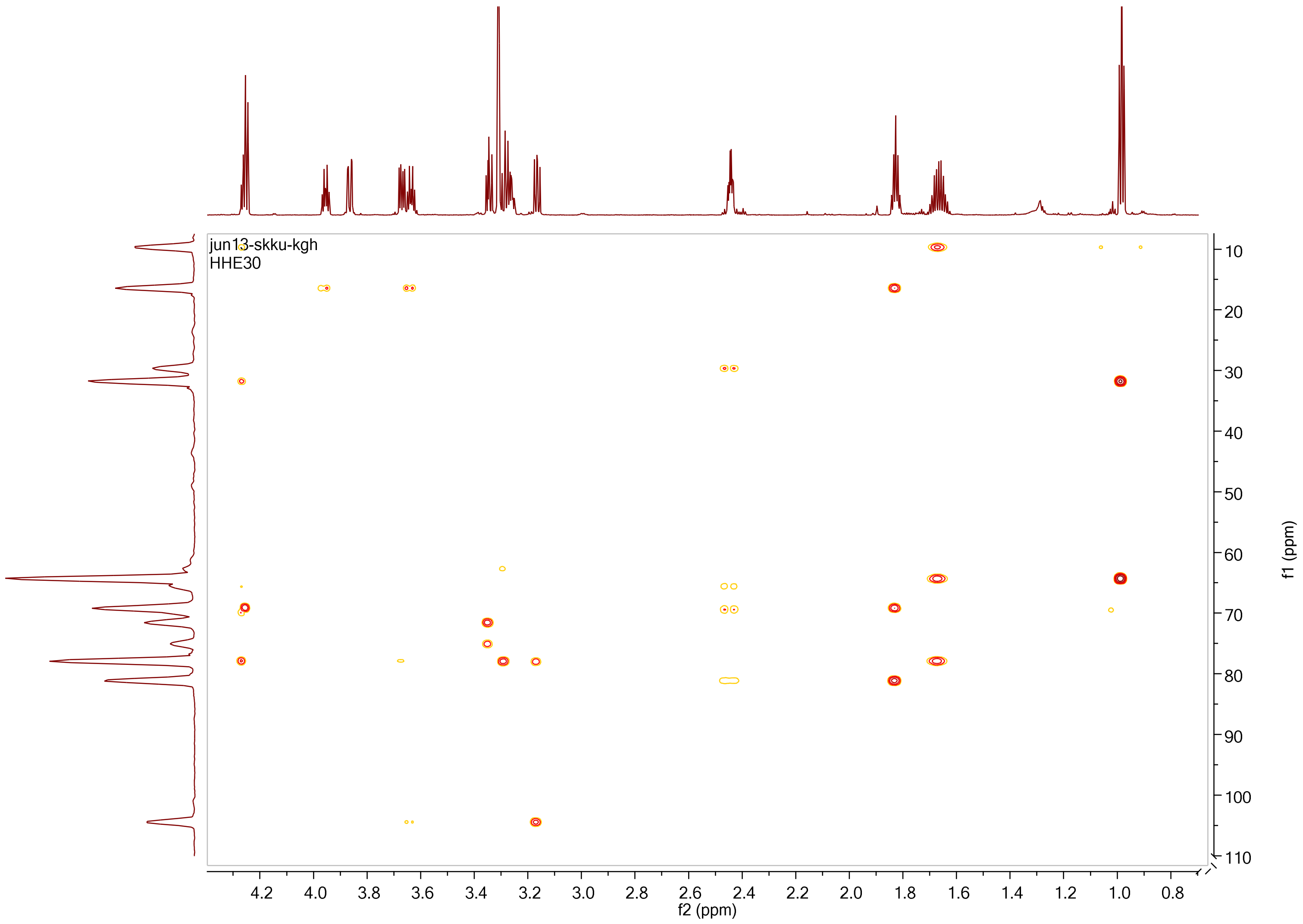

Supplement: Supplementary file 1 [file biomedicines-09-00091-s001.pdf]
